# Preventative and Therapeutic Effects of Low-dose Ionizing Radiation on the Allergic Response of Rat Basophilic Leukemia Cells

**DOI:** 10.1038/s41598-019-52399-9

**Published:** 2019-11-06

**Authors:** Hae Mi Joo, Eun Hee Hong, Seong-Jun Cho, Seon Young Nam, Ji Young Kim

**Affiliations:** Radiation Health Institute, Korea Hydro & Nuclear Power Co., Ltd, Seoul, 132-703 Republic of Korea

**Keywords:** Cell signalling, Signal transduction

## Abstract

The prevalence of allergies has increased over the last four decades. In allergic reactions, mast cells induce a hypersensitive immune response to a substance that is normally harmless. Ionizing radiation has different biological effects depending on the dose and dose rate. In this study, we investigated whether low-dose irradiation before (preventative effect) or after (therapeutic effect) an antigen-antibody reaction has an anti-allergic effect. To test this, we activated rat basophilic leukemia (RBL-2H3) mast cells with anti-2,4-dinitrophenyl IgE (antibody) and 2,4-dinitrophenyl human serum albumin, which served as an antigen. To test for both the potential of a preventative effect and a therapeutic effect, we irradiated mast cells both before and after mast cell activation, and we measured mediator release and signaling pathway activity. Low-dose ionizing radiation suppressed mediator release from RBL-2H3 mast cells activated by the antigen-antibody reaction regardless of when the mast cells were irradiated. These results were due to the suppression of FcεRI expression. Therefore, we suggest that low-dose ionizing radiation has a preventative and therapeutic effect in allergic reactions via the FcεRI-mediated RBL-2H3 mast cell activation system.

## Introduction

According to the survey of World Allergy Organization, about 22 percent of the world’s population suffers from allergic disease^1^. Allergies are harmful immune responses against a large variety of environmental antigens such as pollution, climate change, and pollen^2^. The antigens that enter the body elicit acquired type 2 immune responses by CD4^+^ T helper type (Th2) cells and induced the production of antigen-specific IgE antibodies by B cells^3–5^. In the type 2 immune responses, antigen-specific IgE induce mast cell activation through binding to the high-affinity receptor, FcεRI, for IgE of mast cells^6,7^. When IgE is cross coupled the FcεRI receptor and combined with antigen, the mast cells are activated and release preformed, granule-stored mediators such as histamine, beta-hexosaminidase, and proteases and newly generated mediators such as chemokines, cytokines, and eicosanoids, which enable vascular permeability, smooth muscle constriction, and inflammatory cell recruitment^8–10^. The engagement of FcεRI on mast cells activates multiple signaling pathways and release a various inflammatory mediators result in biological response^11^. The inflammatory mediators secreted from mast cells induce the clinical manifestations of allergic response^11^. The activated pathways induce the recruitment of protein-tyrosine kinases and phosphorylation of various proteins, and Ca^2+^ mobilization^11–13^. Protein-tyrosine kinase such as Lyn, Syk, and Btk^11^ control the degranulation of the mast cells and mitogen-activated protein kinase regulates cytokine expression by FcεRI engagement^14^.

People are exposed to low-dose ionizing radiation every day, including medical diagnostic exposure, occupational exposure, and natural background radiation, in a variety of ways. The biological effects of this low-dose ionizing radiation are quite different from those of high-dose ionizing radiation, but now estimated by extrapolating the effects of high dose radiation of the linear no-threshold (LNT) model^15,16^. While the use of the LNT model has been well established around the world in radiation safety regulations over the past few decades, the scientific community continues to debate the appropriateness of its use^17,18^. Therefore, many international organizations have said that more data on the effects of low-dose ionizing radiation at the molecular, cellular, animal, and human levels are needed.

Ionizing radiation can have different biological effects depending on the dose and dose rate. Some reports claim that low-dose radiation has beneficial effects, but that high-dose radiation is harmful^18,19^. Low-dose whole-body gamma irradiation has been shown to activate immune reactions in several ways, but the effect and mechanism of low-dose radiation on allergic reactions remain poorly understood. In this study, we investigated whether low-dose irradiation, before and after the antibody-antigen reaction, can induce anti-allergic effects.

## Results

### Low-dose ionizing radiation inhibited degranulation and related signaling pathway activity related to IgE-mediated mast cell activation

**Figure 1:**
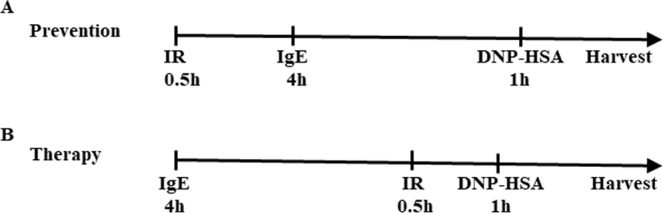
Experimental method of prevention and therapy in the RBL-2H3 mast cell system. (**A**) For a preventative condition RBL-2H3 cells were irradiated and sensitized with DNP-IgE for 4 hr and stimulated with DNP-HAS for 1 hr. (**B**) For a therapeutic condition RBL-2H3 cells were sensitized with DNP-IgE for 4 h and irradiated. After 30 min, cells were stimulated with DNP-HSA for 1 h.

**Figure 2:**
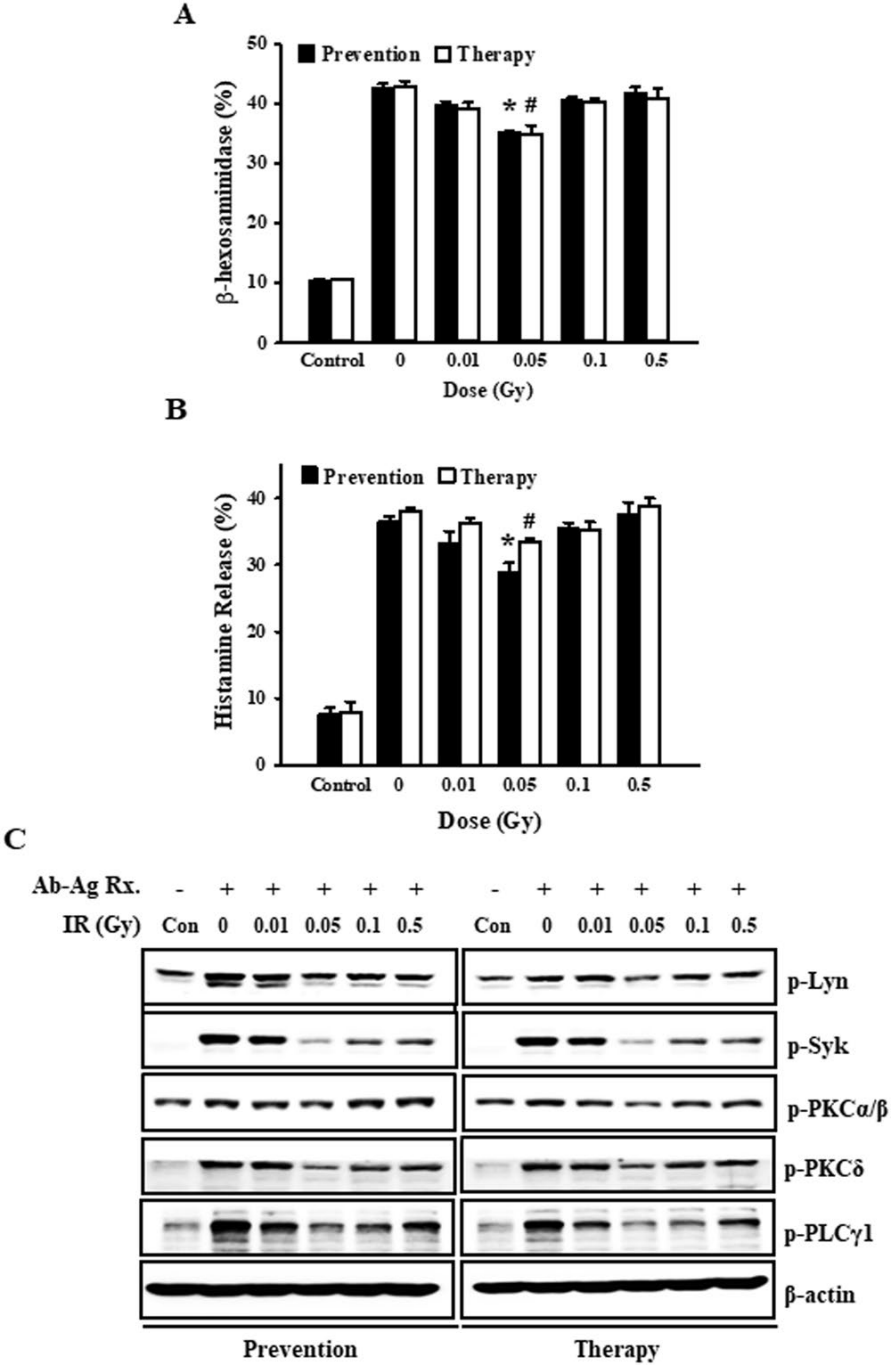
Preventative and therapeutic effects of low-dose ionizing radiation on the degranulation and related signaling pathway. RBL-2H3 cells were irradiated before (preventative effect) and after (therapeutic effect) cells were activated with anti-DNP IgE and stimulated with DNP-HSA for 1 h. Following irradiation, we measured β-hexosaminidase (**A**) and histamine (**B**) in supernatants. (**C**) The phosphorylation of protein tyrosine kinases, p-Lyn, p-Syk, p-PKCα/β, δ, and p-PLCγ1, was detected by western blot analysis following stimulation with DNP-HSA for 10 min. Actin was used as a loading control. Each value represents means ± S.E. for three independent experiments and was analyzed by the *t*-test to determine statistical significance. * and # indicate a value of *p* < 0.05 for the difference between control vs the preventative and therapeutic group, respectively.

To investigate the preventative and therapeutic effects of low-dose ionizing radiation on IgE-mediated mast cell activation, mast cells were irradiated before (to test preventative effect) or after (to test therapeutic effect) they were sensitized by dinitrophenyl (DNP)-immunoglobulin (Ig) E and treated with dinitrophenyl-human serum albumin (DNP-HSA) (Fig. 1). After the antigen-antibody reaction of mast cells was induced, we measured β-hexosaminidase and histamine in the supernatants. As shown Fig. 2A, B, degranulation was increased during IgE-mediated mast cell activation and increased mediator (β-hexosaminidase and histamine) release was remarkably inhibited by 0.05 Gy of low-dose ionizing radiation, regardless of when the mast cells were irradiated. Therefore, our results demonstrated that low-dose ionizing radiation inhibited mast cell degranulation in both the preventative and therapeutic groups.

Crosslinking of FcεRI with IgE and an antigen activates non-receptor-associated protein-tyrosine kinases (PTKs), including Lyn, Syk, PKCs, and PLCγ, and these pathways eventually lead to degranulation^11–13^. Therefore, to determine how low-dose ionizing radiation mediates its preventative and therapeutic effects in mast cell degranulation, we investigated the protein-tyrosine kinase signaling pathway. Phosphorylation of the protein-tyrosine kinases (i.e., Lyn, Syk, PKCs, PLCγ) was increased during IgE-mediated mast cell activation, but increased phosphorylation was gradually reduced by the introduction of 0.01–0.05 Gy radiation, and recovery was observed after 0.1 Gy, like in the degranulation results (Fig. 2C).

### Low-dose ionizing radiation inhibited intracellular free calcium concentration ([Ca^2+^]_i_) in the activated mast cells

**Figure 3:**
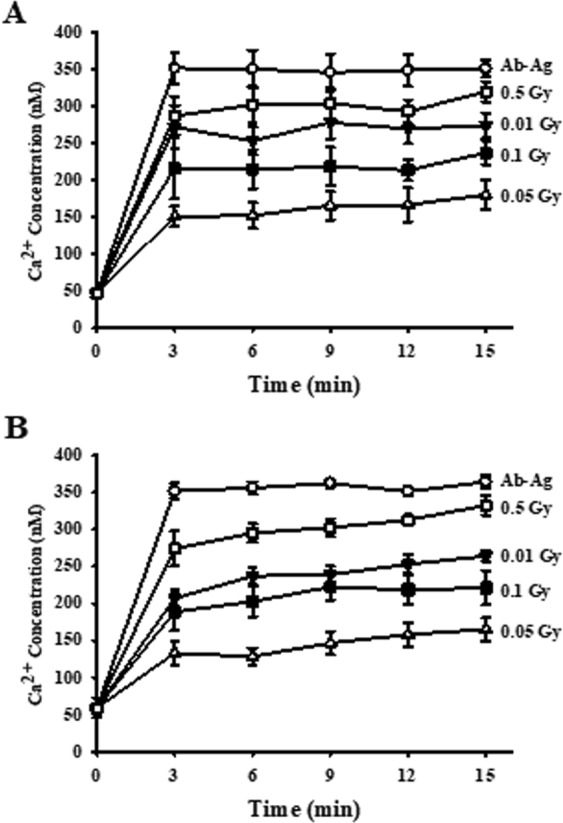
Preventative and therapeutic effects of low-dose ionizing radiation on the intracellular Ca^2+^ mobilization in activated RBL-2H3 cells. RBL-2H3 cells were irradiated before (preventative effect) and after (therapeutic effect) cells were activated with anti-DNP IgE and stained with 5 μM Fluo-3/AM for 30 min. After cells were stimulated with DNP-HAS, Fluo-3/AM-loaded cells were monitored in real time. Each value represents means ± S.E. for three independent experiments, and *t*-test was used to determine statistical significance.

To better understand the suppression mechanism of degranulation by low-dose ionizing radiation, we examined the concentration of intracellular free Ca^2+^ ([C^2+^]_i_) in IgE-mediated mast cell activation. In allergic reactions, [C^2+^]_i_ is elevated. This [C^2+^]_i_ elevation was abolished, however, in the presence of the chelating agent EGTA in the medium, indicating that the calcium influx from the outside of the cells is the major source of [C^2+^]_i_ elevation. To examine the suppression of [C^2+^]_i_ mobilization by low-dose ionizing radiation, we measured [C^2+^]_i_ mobilization by fluometric analysis and found that low-dose ionizing radiation suppressed [C^2+^]_i_ elevation in the IgE-mediated mast cell activation reaction (Fig. 3). In particular, 0.05 Gy irradiation completely blocked [C^2+^]_i_ elevation. This suggests that low-dose ionizing radiation can suppress various intracellular signals associated with degranulation, regardless of the irradiation periods, and that it can have preventative and therapeutic effects.

### Low-dose ionizing radiation inhibited leukotrienes (LTs), cytokines, and related signaling pathway in the activated mast cells

**Figure 4:**
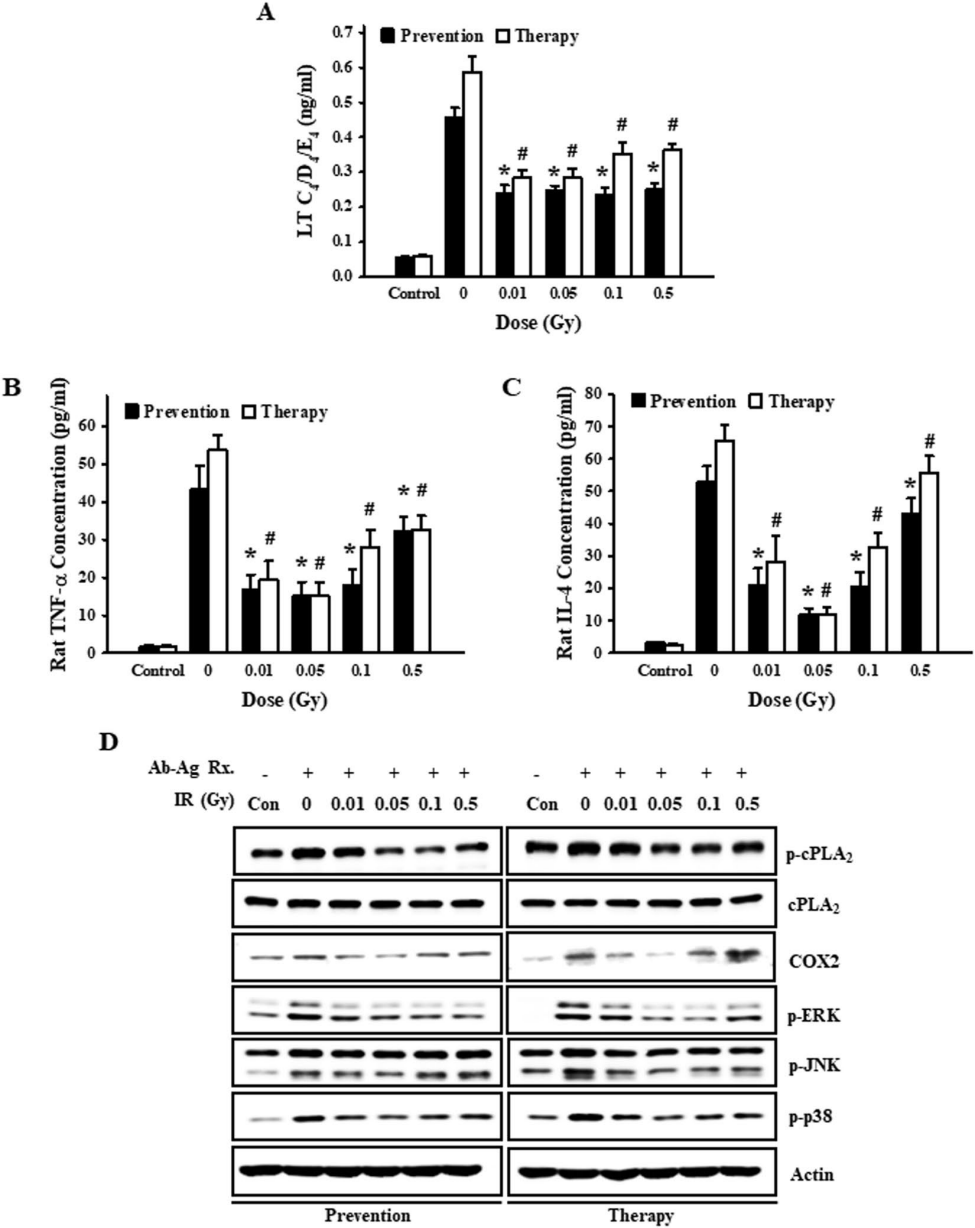
Preventative and therapeutic effects of low-dose ionizing radiation on leukotrienes, cytokines, and the related signaling pathway. We irradiated RBL-2H3 cells before (preventive effect) and after (therapeutic effect) cells were activated with anti-DNP IgE and stimulated with DNP-HSA for 5 h. We then determined leukotrienes (**A**), TNF-α (**B**), and IL-4 (**C**) levels in supernatants. (**D**) Expression of p-cPLA_2_, cPLA_2_, COX, p-ERK, p-JNK, and p-p38 was detected by Western blot analysis following stimulation with DNP-HSA for 10 min. Each value represents means ± S.E. for 3 independent experiments and was analyzed by the *t*-test to determine statistical significance. * and # indicate a value of *p* < 0.05 for the difference between control vs the preventative and therapeutic group, respectively.

Upon stimulation of FcεRI, the mast cells degranulate rapidly, releasing preformed chemical mediators, including histamine and β-hexosaminidase, that play a critical role in the immediate-type allergic response^20^. In contrast, FcεRI activation induces *de novo* synthesis of LTs such as LTC_4_, LTD_4_, LTE_4_, and cytokines, such as TNF-α and IL-4, which initiate the late-phase allergic response^21,22^. To determine the effects of low-dose irradiation on LTs and cytokines secretion, cells were irradiated with different doses either before or after they were sensitized by DNP-IgE and stimulated with DNP-HSA. The results show that release of the LTs (Fig. 4A), TNF-α (Fig. 4B), and IL-4 (Fig. 4C) after FcεRI ligation was significantly increased, but that release was extremely inhibited by low-dose ionizing radiation. Thus, low-dose ionizing radiation is a relatively strong inhibitor of a major mediator of the allergic response.

Cytosolic phospholipase A_2_ (cPLA_2_) is activated by an increase in Ca^2+^ mobilization and ERK activation in the activated mast cells. After activation, cPLA_2_ releases arachidonic acid (AA) from the phospholipid membrane, which is further catalyzed by cyclooxygenase and lipoxygenase and produces, in turn, leukotrienes and prostaglandins^23,24^. Also, 3 major subfamilies of MAPKs (ERK, JNK, and p38) are activated, which results in cytokine production during IgE-mediated mast cell activation^23,24^. To determine the inhibitory mechanism of LTs and cytokine secretion by preventative and therapeutic effects of low-dose ionization, we examined intracellular signaling events (Fig. 4D). Low-dose ionizing radiation with either the preventative or therapeutic protocol inhibited cPLA_2_ phosphorylation and COX2 expression. Also, the phosphorylation of ERK and p38 was strongly suppressed by low-dose ionizing radiation while JNK phosphorylation was weakly controlled (Fig. 4D).

### Low-dose ionizing radiation inhibited FcεRI receptor expression in the mast cells

**Figure 5:**
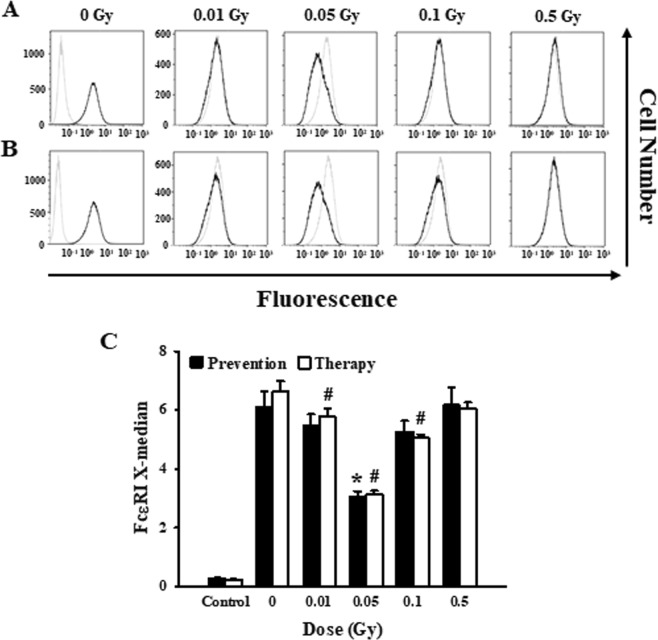
Preventative and therapeutic effects of low-dose ionizing radiation on FcεRI receptor expression. RBL-2H3 cells were irradiated before (preventative effect) (**A**) and after (therapeutic effect) (**B**) cells were activated with IgE and stimulated with FITC-conjugated rat anti-IgE and analyzed by flow cytometry. (**C**) FACS histogram results of (**A**) and (**B**) are presented as a median fluorescence intensity graph. Each value represents means ± S.E. for 3 independent experiments and was analyzed by the *t*-test to determine the statistical significance. * and # indicate a value of *p* < 0.05 for the difference between control vs the preventative and therapeutic group, respectively.

We had previously observed that all mediator release, either performed, granule-stored, or newly generated, was suppressed regardless of which irradiation protocol was used. In addition, each mediator release-related signaling pathway showed the greatest inhibition at 0.05 Gy. We examined the binding affinity of the FcεRI receptor and IgE to ensure that the initial signaling mechanisms induced by antigen-antibody reactions were suppressed by reducing the binding of FcεRI receptors and IgE antibodies in the cell membrane. We found that low-dose ionizing radiation lowered the binding affinity (Fig. 5). This result is consistent with our results for the mediator release, shown earlier, and the signaling pathway control results.

## Discussion

Allergens are recognized by IgE antibodies bound to the FcεRI receptor on the surface of the mast cells^6,7^. Following this recognition, cells release both preformed and newly synthesized mediators of the allergic reaction^8–10^. The therapeutic intervention of allergic disease mainly seeks to block these responses. Recent studies have reported that low-dose irradiation attenuate allergic airway inflammation and tissue remodeling and also used for the clinical treatment of allergies, asthma, rheumatoid arthritis, and other immune disease^25–28^. In previous studies, we suggested that low-dose ionizing radiation suppresses allergic symptoms *in vivo* and inhibits degranulation and inflammatory cytokine expression in the activated mast cell system^29,30^. In this study, we wanted to determine if low-dose ionizing radiation has preventative effects in addition to the established therapeutic effects on allergic reactions.

To confirm this, the time of IgE sensitization was adjusted such that all reactions would be triggered within a day because the irradiation date would vary if IgE antibodies were treated overnight as in previous studies. The appropriate time for cell activity induction via the antigen-antibody reaction of RBL-2H3 mast cells was found to be 4 hours, and the experiment was conducted in the same way as is indicated in Fig. 1.

FcεRI receptor consists of an α chain in which IgE binds, a β chain that has the functions of amplifying signals, and two identical and large intracellular Ɣ chains^31,32^. The signal regions in the form of α-β-Ɣ-Ɣ of FcεRI consist of the following immunoreceptor tyrosine-based activation regions; one is in the β chain and the other is in each of the two Ɣ chains^32,33^.

IgE and FcεRI may be able to induce functional changes directly in FcεRI-bearing cells. FcεRI receptor expression on the mast cell surface can be upregulation dependent on IgE, allowing more IgE to be combined, allowing cells to react with more antigens^34–37^. Therefore, IgE-dependent upregulation of FcεRI receptor can be a large amplification in allergic disease.

Allergens can be cross-linking with IgE bound to mast cells’ FcεRIs, the binding of which triggers the complex signaling events that results in the secretion of a variety of biological active products such as those that are performed and stored in the cells’ cytoplasmic granules (histamine, serotonin, protease, β–hexosaminidase, tryptase). Certain cytokines and lipid-derived mediators like PGD2, LTB4, LTC4, LTD4, and LTE4 are also secreted^32,38–40^.

Antigen ligation of IgE-bound FcεRI elicits phosphorylation of Lyn and Syk, and subsequent recruitment of PLCγ induce the hydrolysis of phosphatidylinositol-4,5-biphosphate, which results in the formation of soluble inositol-1,4,5-triphosphate (IP_3_) and membrane-bound diacylglycerol (DAG). The binding of IP_3_ to its receptor induces calcium mobilization and the PKC signal by DAG to interact synergistically to elicit exocytosis in mast cells^11–13,41^. The activation of the mast cells not only causes the release of preformed granule-associated mediators, but it also initiates *de novo* synthesis of lipid-derived materials. Of these, the cyclooxygenase and lipoxygenase, metabolites of arachidonic acid generated by phospholipase A_2_, has the strongest inflammatory activity^23,24,41^.

A variety of protein-synthesized cytokines produced through the MAP kinase pathway and secreted by activated mast cells during late-phase allergic responses^41^. Degranulation (β–hexosaminidase, histamine), the formation of lipid-derived mediators (LTC4, LTD4, LTE4), and cytokines secretion (TNF-α, IL-4) were inhibited, regardless of whether low-dose ionizing radiation was introduced before or after IgE binding to the FcεRI receptors of mast cells. In addition, the signaling mechanisms associated with the suppression of mediator secretion were all repressed at 0.05 Gy (Figs 2–4). This was thought to be a phenomenon caused by low-dose ionizing radiation inhibiting the initial signal transduction of IgE binding to the FcεRI receptors of mast cells, so we examined the expression change of the FcεRI receptor. As a result, we found that the IgE-FcεRI binding was reduced by low-dose ionizing radiation regardless of the time of irradiation (Fig. 5). We suspect that this reduction is due to the fact that low-dose ionizing radiation temporarily induces three-dimensional structural deformation of the mast cell membrane where the FcεRI receptors exist, thereby inhibiting the environment in which IgE can be combined and crosslinked with the antigen. It is this deformation that is thought to induce the observed therapeutic and preventative effects.

In this study, we have shown that low-dose ionizing radiation inhibited secretion of a variety of mediators. The results from the initial suppression of the signaling pathway (Figs 2–4), via the reduction of FcεRI receptor expression (Fig. 5), are now part of a well-established therapeutic and preventative experimental system. Finally, we have demonstrated here, for the first time, both the preventative and therapeutic effects of low-dose ionizing radiation against IgE-mediated mast cell activation. These effects may be useful for the identification of benefits of low-dose ionizing radiation, which could lead to a better treatment response in allergic disease.

## Methods

### Cell culture

Rat basophilic leukemia (RBL-2H3) cells were purchased from the American Type Culture Collection (ATCC, Manassas, VA). The cells were cultured in Eagle’s minimum essential medium (EMEM, GIBCO, Grand Island, NY) containing 15% v/v fetal bovine serum (GIBCO, Grand Island, NY), and they were maintained at 37 °C in a humidified incubator containing 5% CO_2_^29,30^.

### Cell irradiation

RBL-2H3 cells were irradiated with 0.01–0.5 Gy using a ^137^Cs-γ-irradiator (Gammercell^®^ 40 Exactor, Best Theratronics, Ltd., Ottawa, Canada) with a dose rate of 0.5 Gy/min^29,30^. The irradiation was performed according to the protocol given in Fig. 1.

### RBL-2H3 cell activation

RBL-2H3 cells were sensitized with 0.1 µg/mL monoclonal anti-dinitriphenyl (DNP) (IgE) antibody clone SPE-7 (Sigma, St. Louis, MO). Cells were washed with modified Tyrode’s buffer (TGCM buffer) consisting of 137 mM NaCl, 0.42 mM NaH_2_PO_4_, 2.6 mM KCl, 1 mM CaCl_2_, 0.5 mM MgCl_2_, 12 mM NaHCO_2_, 5 mM dextrose, 1 g/L glucose, and 1 µg/L gelatin with a pH 7.4. Cells were stimulated with 0.01 µg/mL DNP-human serum albumin (HAS) (Sigma, St. Louis, MO)^29,30^.

### Assay for histamine and β-hexosaminidase

After 1 hour antigen-antibody reaction, histamine concentration was detected using enzyme immunoassay for histamine (Oxford Biomedical Research, Rochester Hills, MI). The amount of released histamine was expressed as a percentage of the total histamine produced by unstimulated cells. To determine β-hexosaminidase release, supernatants and lysed pellets were aliquoted into 96-well plates, and samples were mixed with substrate solution [1 mM p-nitrophenyl N-acetyl-beta-D-glucosamine in 0.05 M sodium carbonate buffer (pH 10)]. Absorbance was measured with a spectrophotometer (Labsystems, Helsinki, Finland) at 405nm^29,30^.

### Cytokine measurement

IgE-sensitized RBL-2H3 cells were irradiated and stimulated with DNP-HAS for 5 h. Interleukin-4 (IL-4) and tumor necrosis factor-α (TNF-α) concentrations in cell culture supernatants were measured using enzyme-linked immunosorbent assay (ELISA) kits (R&D Systems Inc., Minneapolis, MN)^29,30^.

### Leukotrienes (LTC_4_/D_4_/E_4_) measurement

IgE-sensitized RBL-2H3 cells were irradiated and stimulated with DNP-HAS for 5 h. Leukotrienes (LTC_4_/D_4_/E_4_) concentrations in cell culture supernatants were measured using enzyme-linked immunosorbent assay (ELISA) kits (Neogen Corp., Lansing, MI).

### Measurement of intracellular Ca^2+^ levels

RBL-2H3 cells were sensitized, irradiated, and incubated with 5 μM Fluo-3/AM (Molecular Probes, Eugene, OR) in TGCM buffer (without CaCl_2_) for 30 min at 37 °C. After washing, cells were stimulated with DNP-HAS. Fluo-3/AM fluorescence intensities were monitored using a microplate fluorometer (Berthold Technologies, Bad Wildbad, Germany). Cytosolic free calcium concentration ([Ca^2+^]_i_) were calculated as previously described^25,29,30^.

### Immunoblotting

RBL-2H3 cells were harvested and lysed in a lysis buffer containing 20 mM Tris (pH 7.6), 150 mM NaCl, 1% Triton X-100, protease inhibitors cocktail, and phosphatase inhibitor cocktail (Thermo Scientific, Waltham, MA). Protein quantification was determined by Bradford assay (Bio-Rad, Hercules, CA). The cell lysates of equal protein concentrations were prepared in LDS sample buffer (Thermo Scientific, Waltham, MA), separated on SDS-PAGE (10% or 12% acrylamide), and transferred to nitrocellulose membranes (Amersham Biosciences, Arlingston Heights, IL). Membrane were blocked 5% skim milk (GIBCO, Grand Island, NY) in PBS containing 0.1% Tween 20, followed by incubation with the indicated antibodies for 3 hr at room temperature or overnight at 4 °C. After washing with PBS containing 0.1% Tween 20, membranes were incubated with secondary antibodies for 1 hr at room temperature. Protein bands were visualized using the ECL solution (Amersham Biosciences, Bath, UK)^29,30^.

### Flow cytometry

RBL-2H3 cells were sensitized and irradiated with 0.01–0.5 Gy. After incubating 30 min, cells were treated with FITC-conjugated rat anti-IgE antibodies (BD Pharmingen, San Diego, CA) and analyzed by flow cytometry (Beckman Coulter Inc., Krefeld, Germany)^29,30^.

### Statistics analysis

Experimental data are represented as means ± S.E. Statistical analysis (*t*-test) were performed using SAS software (version 8.1; SAS Institute., Cary, NC). *p*-value less than 0.05 was considered statistically significant.
